# Association of Gestational Vitamin E Status With Pre-eclampsia: A Retrospective, Multicenter Cohort Study

**DOI:** 10.3389/fnut.2022.911337

**Published:** 2022-06-21

**Authors:** Huifeng Shi, Yuanhui Jiang, Pengbo Yuan, Lian Chen, Xiaoli Gong, Yike Yang, Yuanyuan Wang, Hai Jiang, You Li, Mengxing Sun, Yangyu Zhao, Yuan Wei

**Affiliations:** ^1^Department of Obstetrics and Gynecology, Peking University Third Hospital, Beijing, China; ^2^National Clinical Research Centre for Obstetrics and Gynecology, Beijing, China; ^3^National Centre for Healthcare Quality Management in Obstetrics, Beijing, China

**Keywords:** vitamin E, pregnancy, pre-eclampsia, gestational change, cohort

## Abstract

**Introduction:**

Pre-eclampsia is the second leading cause of maternal mortality worldwide. The controversy for the association of vitamin E with pre-eclampsia has raged unabated for two decades. We aimed to determine the association of vitamin E level in the first trimester and the gestational change with pre-eclampsia.

**Materials and Methods:**

A retrospective cohort study was conducted among singleton pregnant women aged 15–49 years at 137 hospitals in China. Serum vitamin E concentrations in the first trimester and at pre-eclampsia assessment time were uniformly quantified in a laboratory by high performance liquid chromatography. Logistic regression models with restricted cubic splines were performed to reveal a non-linear association of vitamin E concentrations in the first trimester and the gestational change with pre-eclampsia.

**Results:**

We included 73 317 participants (47.8% aged 25–29 years) and 2.28% were diagnosed with pre-eclampsia. Higher risk was observed in those with lower concentration in the first trimester and greater gestational decrease, with a range from 0.81 to 80.60%. A non-linear L-shaped association was observed between vitamin E concentrations in the first trimester and pre-eclampsia, suggesting a threshold at 7.3 mg/L and a ceiling effect: the risk saw a steep rise when the concentrations in the first trimester were < 7.3 mg/L but was relatively flat beyond the inflection point. Sharply increased pre-eclampsia risk was also found in those with gestational vitamin E decrease after accounting for the baseline status in the first trimester. However, gestational vitamin E increase was associated with decreased pre-eclampsia risk when the baseline concentrations were < 7.3 mg/L but did not confer additional benefits when it was above the threshold.

**Conclusion:**

We demonstrated alarmingly high pre-eclampsia risk in women with vitamin E concentrations of < 7.3 mg/L in the first trimester and gestational vitamin E decrease. These findings underscore the need to supplement vitamin E among pregnant women with low baseline status.

## Introduction

Pre-eclampsia is a multisystem complication marked by the gestational onset of hypertension and proteinuria (1). It complicates 4.6% of pregnancies (2), and is the second leading cause of maternal mortality worldwide, accounting for approximately 30 000 maternal deaths annually, mostly in low- and middle-income countries (3, 4). Pre-eclampsia can also cause other severe maternal and perinatal complications and even fetal mortality (5). In China, pre-eclampsia affects 2–3% of pregnancies and leads to about 10% of maternal death (6, 7). Given no known cure for pre-eclampsia other than delivery, identifying the risk factors and developing prevention strategies is critical for reducing the incidence of pre-eclampsia and achieving a lower level of maternal and fetal mortality and morbidity.

The etiology of pre-eclampsia remains largely unknown, but studies suggest that oxidative stress may be implicated in the pathogenesis (8). Several antioxidant vitamins, such as vitamin E, have been proposed by cytological study and animal experiments to have an important role in the cause and prevention of pre-eclampsia (9–14). However, the controversy about scientific evidence for the association has also raged unabated for two decades (15–19), despite several randomized controlled trials (RCTs) (20–31). Additionally, it deserves attention but a paucity of evidence remains on the impacts of vitamin E baseline status and relative change during pregnancy on pre-eclampsia.

There is no available data on the global prevalence of vitamin E deficiency or excess. A cross-sectional study, which included 119,286 women from 17 cities in 4 provinces in western China between 2017 and 2019, found that the reference range was 7.4–23.5 mg/L for vitamin E during pregnancy, with 2.5% of pregnant women below and 2.5% above the limits (32). Given so many pregnant women with vitamin E deficiency or excess, identifying their risk of pre-eclampsia is warranted. This study aimed to determine the association of vitamin E levels in the first trimester and the relative change during pregnancy with the risk of pre-eclampsia through a retrospective, multicenter, large-sample cohort.

## Materials and Methods

### Study Setting and Population

We conducted a retrospective cohort study with a secondary analysis using data from a prospective cohort investigating vitamin E concentrations during pregnancy. The prospective cohort study was conducted at 180 maternity hospitals in 23 provinces in China between 2015 and 2018. Pregnant women who met the following criteria were recruited to participate: at age of 15–49 years, singleton pregnancy, and planning to be registered for antenatal care and deliver in these hospitals. Eligible women who agreed to participate were followed up for assessing their health conditions and collecting blood samples at least once during pregnancy.

**FIGURE 1:**
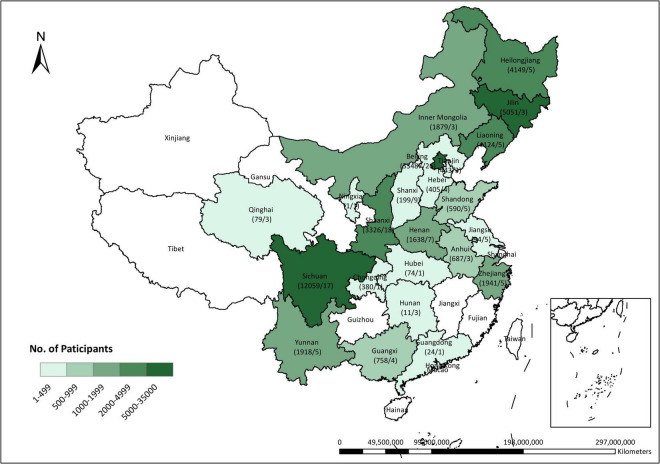
Distribution of participants across China. The numerator denotes the number of pregnant women who were included in this study cohort and the denominator denotes the number of hospitals as the research centers recruiting the participants.

According to the study objectives, our analyses only included singleton pregnant women aged 15–49 years with pre-eclampsia assessment at 20–40 weeks and available data of vitamin E concentrations in the first trimester (at 4–13 weeks of gestation) and at the time of pre-eclampsia assessment. Women with prepregnancy hypertension were excluded. Finally, 73 317 pregnant women with required data from 137 hospitals were included (Figure 1 and Supplementary Figure 1). All process of this study was reviewed and approved by the Peking University Third Hospital Medical Science Research Ethics Committee (IRB00006761–2015277). Written informed consent was obtained from all participants at the time of recruitment.

### Gestational Vitamin E Status

(1) *Laboratory procedures and analyses*. At each follow-up, a fasting venous blood sample (2 ml) was collected in a vacuum tube and then centrifuged within 1 h by trained nurse technicians under no-light condition. Serum was extracted and aliquoted immediately into eppendorfs, protected from light, and stored at –80°C before being sent to the Central (Laboratory of Beijing Harmony Health Medical Diagnostics Co., Ltd., Beijing, China) on dry ice (within 1 months of blood drawn) and maintained at –80°C until analyzed. Within 24 h after receiving samples, serum concentrations of vitamin E (α-tocopherol) were then quantified by using high performance liquid chromatography (HPLC) (Prominence LC-20A,Shimadzu) in a dark room. Duplicate analyses were performed on at least one-twentieth of each batch of samples and the interassay coefficients of variation were < 5%. The within- and between-day relative standard deviations of the controls over the course of analysis were 3.86 and 4.52% for measuring α-tocopherol concentrations, respectively.

(2) *Calculation of vitamin E level*. We calculated each participant’s vitamin E levels in the first trimester and at the gestational age of pre-eclampsia assessment. The minimum vitamin E concentration was used to represent the level in the first trimester if it was measured twice or more during the gestational period. The vitamin E level at the time of pre-eclampsia assessment was represented by the concentration at the last assessment time if all pre-eclampsia assessments (≥1 times) were negative or at the time of being firstly diagnosed with pre-eclampsia. Gestational change of the vitamin E concentration was calculated by subtracting the concentration in the first trimester from the concentration at the time of pre-eclampsia assessment which was then divided by the concentration in the first trimester and the result was taken out in percentage terms.

### Diagnosis on Pre-eclampsia

Pre-eclampsia was diagnosed with the criteria recommended by the Chinese Society of Obstetrics and Gynecology as follows: new onset hypertension (systolic ≥ 140 mmHg and/or diastolic ≥ 90 mmHg) at or after 20 weeks of gestation, accompanied by one or more other features: proteinuria (dipstick ≥ 1 +, random protein/creatinine ratio ≥ 30 mg/mmol or 0.3 g/24 h), other maternal organ dysfunction (including heart, lung, liver, kidney), or hematological, digestive, and neurological involvement, and/or uteroplacental dysfunction (33).

### Covariates

We extracted geographical region, age, ethnic origin (Han or others), education, Hukou (urban residents, rural residents, or rural-to-urban migrants), mode of conception (Assisted reproductive technology [ART] or conceiving naturally), primigravida, and pre-pregnancy body mass index (BMI) (being classified as underweight [<18.5], normal BMI [18.5–23.9], overweight [24–27.9], obesity [≥28] or unknown by using the diagnostic criteria in Chinese adults (34)), and diabetes from medical records.

### Statistical Analysis

Vitamin E concentrations in the first trimester were divided into eight groups (<5.5, 5.5–6.4, 6.5–7.2, 7.3–8.0, 8.1–9.5, 9.6–11.4, 11.5–13.9, and 14.0–35.9 mg/L) according to the 1st, 2.5th, 5th, 10th, 25th, 50th, and 75th percentiles in all pregnant women enrolled in the original cohort (Supplementary Table 1). According to the corresponding 10th, 25th, 50th, 75th, and 90th percentiles, gestational change of the vitamin E concentration was divided into six groups (≤-10, –9 to –1, 0–29, 30–59, 60–89, and ≥ 90%). Absolute risks were calculated as the percentage of women with pre-eclampsia within each combination of vitamin E concentration categories in the first trimester and gestational change categories.

The associations of vitamin E concentrations in the first trimester and the gestational change with pre-eclampsia were examined by performing logistic regression models with restricted cubic splines. Vitamin E concentrations in the first trimester and the gestational change were modeled using restricted cubic splines to allow for more flexibility of the association and knots were determined according to the principle of minimized Akaike Information Criterion (AIC) (35). Sensitivity analyses were performed by adjusting for different covariates in the multivariable models. In model A, we adjusted for no covariate expect for the confounding of the concentrations in the first trimester and the gestational change on each other. In model B, we additionally adjusted for aforementioned covariates including age, education, ethnic origin, geographical region, Hukou, ART, primigravida, pre-pregnancy BMI groups, and diabetes. Predicted absolute probabilities of pre-eclampsia with 95% confidence intervals (CIs) were calculated with respect to vitamin E concentrations in the first trimester. Additionally, by these models, we determined the association of gestational change of vitamin E concentrations with pre-eclampsia with respect to varied vitamin E concentrations in the first trimester (1st, 2.5th, 5th, 10th, 25th, 50th, 75th, 90th, 95th, 97.5th, and 99th). Similar analyses as model B were also conducted in pregnant women grouped by gestational weeks of pre-eclampsia assessment (20–23, 24–27, 28–30, 31–33, 34–36, and 37–40 weeks) to identify the association of vitamin E concentrations in the first trimester and the gestational change with the risk of pre-eclampsia at different stages of pregnancy. Another reason why this subgroup analysis was performed is that both the occurrence of pre-eclampsia and gestational vitamin E level are associated with gestational age, which may bias the estimation about the association of gestational vitamin E level and the changes with preeclampsia.

Furthermore, we employed robust Poisson regression to assess the association of grouped vitamin E concentrations in the first trimester with pre-eclampsia. Sensitivity analyses were performed by adjusting for different covariates. In Model 1, we adjusted aforementioned covariates. In model 2, we additionally adjusted for gestational vitamin E change categories. Model 3 additionally included an interaction term of grouped vitamin E concentrations in the first trimester and the gestational change based on Model 2. We also performed a stratified analysis by grouped vitamin E concentrations in the first trimester. In each group, the association of gestational vitamin E change categories with pre-eclampsia was assessed by using robust Poisson regression models that adjusted for aforementioned covariates. All associations were presented by relative risks (RRs) and the 95% CIs which were calculated in these models.

All statistical analyses were performed with SAS software, version 9.0 and the R statistical software, version 3.6.2. A two-tailed *p*-value < 0.05 was considered statistically significant.

## Results

Among 73 317 pregnant women (47.8% at age of 25–29 years) included in the study, 2.28% (1675 cases) were diagnosed with pre-eclampsia (Table 1).

**TABLE 1:** Basic characteristics of participants.

	Non-Pre-eclampsia (*n* = 71642)	Pre-eclampsia (*n* = 1675)	Total (*n* = 73317)
**Region, No. (%)**			
North China	35474 (49.5)	939 (56.1)	36413 (49.7)
Northeast China	12916 (18.0)	408 (24.4)	13324 (18.2)
East China	3305 (4.6)	7 (0.4)	3312 (4.5)
Central-south China	2470 (3.4)	35 (2.1)	2505 (3.4)
Southwest China	14108 (19.7)	249 (14.9)	14357 (19.6)
Northwest China	3369 (4.7)	37 (2.2)	3406 (4.6)
**Age group (years), No. (%)**			
15–19	414 (0.6)	10 (0.6)	424 (0.6)
20–24	9159 (12.8)	172 (10.3)	9331 (12.7)
25–29	34337 (47.9)	726 (43.3)	35063 (47.8)
30–34	20037 (28.0)	536 (32.0)	20573 (28.1)
35–39	6680 (9.3)	192 (11.5)	6872 (9.4)
40–49	1015 (1.4)	39 (2.3)	1054 (1.4)
**Ethnic origin, No. (%)**			
Han	70283 (98.1)	1651 (98.6)	71934 (98.1)
Other	1359 (1.9)	24 (1.4)	1383 (1.9)
**Education, No. (%)**			
High school	26999 (37.7)	676 (40.4)	27675 (37.7)
College	22353 (31.2)	661 (39.5)	23014 (31.4)
Master	4367 (6.1)	89 (5.3)	4456 (6.1)
Other	17923 (25.0)	249 (14.9)	18172 (24.8)
**Hukou, No. (%)**			
Urban residents	58193 (81.2)	1416 (84.5)	59609 (81.3)
Migrants	4804 (6.7)	122 (7.3)	4926 (6.7)
Rural residents	8645 (12.1)	137 (8.2)	8782 (12.0)
**Pre pregnancy BMI, No. (%)**			
Underweight [<18.5]	9686 (13.5)	171 (10.2)	9857 (13.4)
Normal BMI [18.5–23.9]	50297 (70.2)	921 (55.0)	51218 (69.9)
Overweight [24–27.9]	8916 (12.4)	347 (20.7)	9263 (12.6)
Obesity [≥28]	2257 (3.2)	224 (13.4)	2481 (3.4)
Unknown	486 (0.7)	12 (0.7)	498 (0.7)
**Primigravida, No. (%)**	38826 (54.2)	917 (54.7)	39743 (54.2)
**ART, No. (%)**	486 (0.7)	35 (2.1)	521 (0.7)
**Diabetes, No. (%)**	88 (0.1)	4 (0.2)	92 (0.1)

### Pre-eclampsia Risk by Vitamin E Concentrations in the First Trimester

**FIGURE 2:**
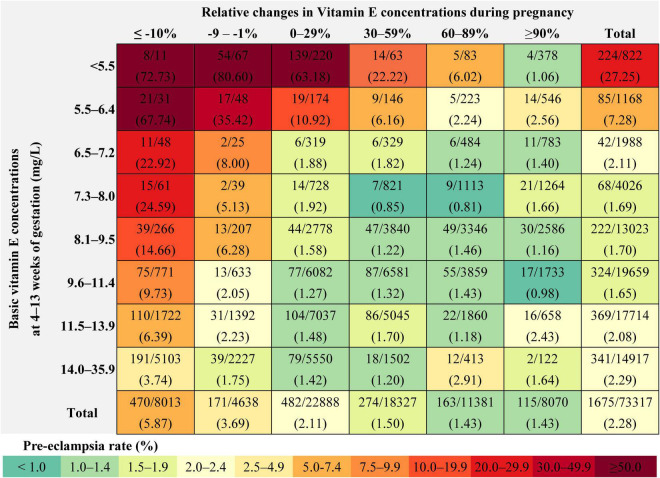
Rate of pre-eclampsia within each combination of the vitamin E concentration categories in the first trimester and the gestational change categories.

The absolute risk for pre-eclampsia was 27.25, 7.28, 2.11, 1.69, 1.70, 1.65, 2.08, and 2.29% among pregnant women with vitamin E concentrations of < 5.5 (<1st), 5.5–6.4 (1st–2.4th), 6.5–7.2 (2.5th–4th), 7.3–8.0 (5th–9th), 8.1–9.5 (10th–24th), 9.6–11.4 (25th–49th), 11.5–13.9 (50th–74th), and 14.0–35.9 (75th–100th) mg/L in the first trimester, respectively (Figure 2).

**FIGURE 3:**
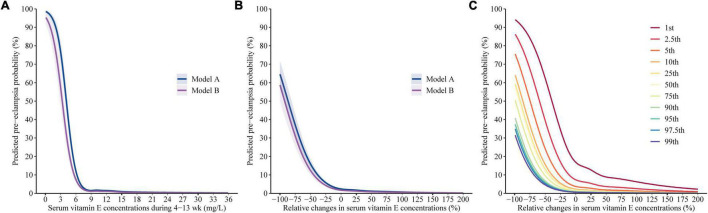
Predicted absolute risks of pre-eclampsia with respect to gestational vitamin E status. Predicted absolute risks of pre-eclampsia with 95% CIs were calculated with respect to **(A)** the vitamin E concentrations in the first trimester and **(B)** the gestational change by performing logistic regression models with restricted cubic splines. Model A adjusted for the confounding of the concentrations in the first trimester and the gestational change on each other. Model B additionally adjusted for age, education, ethnic origin, region, Hukou, ART, primigravida, pre pregnancy BMI, and diabetes. **(C)** Predicted absolute risks of pre-eclampsia calculated by using Model B with respect to gestational vitamin E change given varied baseline concentrations (1st, 2.5th, 5th, 10th, 25th, 50th, 75th, 90th, 95th, 97.5th, and 99th percentiles) in the first trimester.

Using restricted cubic spline regression analysis, we found that the association of vitamin E concentrations in the first trimester with the risk of pre-eclampsia is presented as an L-shaped curve with an inflection point at 7.3 mg/L (the predicted probability of pre-eclampsia was 2.4% at the point which was roughly equal to the estimated prevalence [2.3%] in China). There was a steep rise in the risk for women whose vitamin E concentrations in the first trimester were less than 7.3 mg/L; However, risk was relatively flat beyond the inflection point (Figure 3A). Adjusting for different covariates did not substantially influence the estimates. The similar association was also observed in subgroup analyses stratified by gestational weeks of pre-eclampsia assessment (Supplementary Figure 2).

The results of multivariable adjusted robust Poisson regression show that compared with women with vitamin E concentrations of 9.6–11.4 mg/L (25th–49th) in the first trimester, those with vitamin E concentrations of < 5.5 mg/L and 5.5–6.4 mg/L in the first trimester had 29.56-fold (95%CI, 22.92–38.12) and 7.42-fold (95% CI, 4.78–11.51) increased risks for pre-eclampsia, respectively (Table 2).

**TABLE 2:** Adjusted relative risks (95% CI) for pre-eclampsia according to vitamin E concentrations in the first trimester.

	Model 1^a^		Model 2^b^		Model 3^c^	
	aRR (95% CI)	*p*-value	aRR (95% CI)	*p*-value	aRR (95% CI)	*p*-value
**Vitamin E concentrations at 4–13 weeks of gestation (mg/L)**						
<5.5	**10.97 (9.38, 12.83)**	**<0.001**	**12.79 (10.91, 14.99)**	**<0.001**	**29.56 (22.92, 38.12)**	**<0.001**
5.5–6.4	**4.01 (3.22, 5.01)**	**<0.001**	**4.74 (3.85, 5.82)**	**<0.001**	**7.42 (4.78, 11.51)**	**<0.001**
6.5–7.2	1.33 (0.97, 1.83)	0.077	**1.69 (1.24, 2.29)**	**0.001**	1.51 (0.67, 3.43)	0.319
7.3–8.0	1.06 (0.82, 1.37)	0.676	**1.37 (1.06, 1.77)**	**0.016**	1.52 (0.87, 2.65)	0.142
8.1–9.5	1.05 (0.89, 1.25)	0.535	**1.24 (1.05, 1.46)**	**0.013**	1.27 (0.88, 1.83)	0.206
9.6–11.4	1.00 [Reference]		1.00 [Reference]		1.00 [Reference]	
11.5–13.9	**1.25 (1.08, 1.45)**	**0.003**	0.97 (0.84, 1.13)	0.727	1.15 (0.86, 1.54)	0.351
14.0–35.9	**1.37 (1.18, 1.60)**	**<0.001**	**0.64 (0.54, 0.76)**	**<0.001**	1.10 (0.80, 1.50)	0.558

*^a^Model 1 adjusted for age, education, ethnic origin, geographical region, Hukou, ART, primigravida, pre pregnancy BMI groups, and diabetes.*

*^b^Model 2 additionally adjusted for gestational vitamin E change categories.*

*^c^Model 3 additionally included an interaction term of grouped vitamin E concentrations in the first trimester and the gestational change based on Model 2.*

*Bold number refers to the value of RRs with 95% CIs significantly lower than 1.*

### Pre-eclampsia Risk by Gestational Vitamin E Change

The absolute risk for pre-eclampsia was 5.87, 3.69, 2.11, 1.50, 1.43, and 1.43% among pregnant women with gestational change of ≤ –10, –9 to –1, 0–29, 30–59, 60–89, and ≥ 90% in vitamin E concentrations before pre-eclampsia assessment, respectively. The absolute risk ranged from 0.81 to 80.60% for pre-eclampsia in all combinations of the vitamin E concentration categories in the first trimester and the gestational change categories; higher risk was observed in those with lower concentration in the first trimester and greater decrease during pregnancy. In pregnant women with vitamin E concentration of < 5.5 mg/L in the first trimester, the incidence of pre-eclampsia was 72.73, 80.60, 63.18, 22.22, 6.02, and 1.06% in those with gestational change of ≤ –10, –9 to –1, 0–29, 30–59, 60–89, and ≥ 90% in vitamin E concentrations before pre-eclampsia assessment, respectively. This value was 67.74, 35.42, 10.92, 6.16, 2.24 and 2.56%, respectively in pregnant women with vitamin E concentration of 5.5–6.4 mg/L in the first trimester and corresponding gestational changes (Figure 2).

Figures 3B,C indicated clear L-shaped trends of the predicted risks of pre-eclampsia across gestational vitamin E change; Figure 3C additionally presented that the inflection points at the curves decreased by the vitamin E concentrations in the first trimester. The inflection point (where the predicted probability of pre-eclampsia was equal to the estimated prevalence [2.3%] in China) was gestational vitamin E change of 197, 110, 34, –2, –9, –10, –17, –26, –29, –31, and –35% among women with the vitamin E concentration of 5.5, 6.5, 7.3, 8.1, 9.6, 11.5, 14.0, 16.9, 19.0, 21.1, and 23.9 mg/L in the first trimester, respectively. In pregnant women with vitamin E concentrations of < 7.3 mg/L (5th) in the first trimester, a steep rise in the risk of pre-eclampsia was observed along with a decrease in gestational vitamin E concentration and gestational vitamin E increase was associated with decreased risk of pre-eclampsia. In pregnant women with vitamin E concentrations of ≥ 7.3 mg/L in the first trimester, the inflection point was the relative change degree of ≤ 0% and risk was relatively flat along with gestational vitamin E increase. The similar L-shaped association of gestational vitamin E change with pre-eclampsia was also observed in subgroup analyses stratified by gestational weeks of pre-eclampsia assessment (Supplementary Figure 2).

Multivariable adjusted robust Poisson regressions show similar associations (Table 3). Within each group of the vitamin E concentration categories in the first trimester except for the group of < 5.5 mg/L, gestational vitamin E decrease of ≥ 10% was significantly associated with 2.3–11.6fold increased risks for pre-eclampsia, compared with gestational vitamin E increase of 0–29%. The corresponding aRRs were more than 7.5 in women with vitamin E concentrations of 6.5–11.4 mg/L in the first trimester. In women with vitamin E concentrations of < 6.5 mg/L in the first trimester, gestational vitamin E increase of ≥ 60% was significantly associated with 60–90% decrease in risks for pre-eclampsia, compared with gestational vitamin E increase of 0–29%. In women with vitamin E concentrations of 7.3–8.0 mg/L in the first trimester, gestational vitamin E increase of 30–59 and 60–89% was significantly associated with 60% (aRR: 0.40 [95% CI 0.17–0.97]) and 66% (aRR: 0.34 [95% CI 0.15–0.79]) decrease in risks for pre-eclampsia, respectively, compared with gestational vitamin E increase of 0–29%. In women with vitamin E concentrations of ≥ 8.1 mg/L in the first trimester, no significant decrease in risk for pre-eclampsia was found among those with gestational vitamin E increase of ≥ 30% compared with gestational increase of 0–29%.

**TABLE 3:** Adjusted relative risks (95% CI) for pre-eclampsia according to gestational vitamin E concentration changes stratified by vitamin E concentration categories in the first trimester.

		Relative changes of vitamin E concentrations during pregnancy					
	*N*	≤-10%	-9 to -1%	0–29%	30–59%	60–89%	≥90%
**Basic vitamin E concentrations at 4–13 weeks of gestation (mg/L)**							
<5.5^a^	822	1.11 (0.99, 1.24)	1.10 (0.97, 1.25)	1.00 [Reference]	0.79 (0.59, 1.06)	**0.44 (0.20, 0.93)**	**0.11 (0.04, 0.30)**
5.5–6.4^a^	1168	**2.28 (1.50, 3.46)**	**1.75 (1.17, 2.64)**	1.00 [Reference]	0.62 (0.34, 1.15)	**0.33 (0.13, 0.86)**	**0.32 (0.18, 0.60)**
6.5–7.2^a^	1988	**7.95 (2.87, 22.04)**	3.23 (0.72, 14.62)	1.00 [Reference]	0.76 (0.23, 2.56)	0.50 (0.15, 1.64)	0.60 (0.20, 1.83)
7.3–8.0^a^	4026	**11.62 (6.06, 22.29)**	2.88 (0.63, 13.09)	1.00 [Reference]	**0.40 (0.17, 0.97)**	**0.34 (0.15, 0.79)**	0.71 (0.36, 1.40)
8.1–9.5^a^	13023	**7.55 (5.02, 11.34)**	**3.52 (1.94, 6.38)**	1.00 [Reference]	0.69 (0.46, 1.05)	0.80 (0.53, 1.22)	0.64 (0.40, 1.03)
9.6–11.4^a^	19659	**7.57 (5.49, 10.43)**	1.62 (0.90, 2.90)	1.00 [Reference]	0.98 (0.72, 1.34)	1.04 (0.74, 1.48)	0.74 (0.44, 1.24)
11.5–13.9^a^	17714	**4.25 (3.25, 5.57)**	1.46 (0.98, 2.18)	1.00 [Reference]	1.13 (0.85, 1.50)	0.79 (0.50, 1.25)	1.54 (0.91, 2.60)
14.0–35.9^a^	14917	**2.48 (1.89, 3.26)**	1.28 (0.87, 1.90)	1.00 [Reference]	1.08 (0.65, 1.82)	**2.69 (1.47, 4.95)**	1.70 (0.42, 6.96)
Total^b^	73317	**7.42 (5.45, 10.10)**	1.60 (0.89, 2.85)	1.00 [Reference]	1.02 (0.75, 1.39)	1.08 (0.77, 1.53)	0.77 (0.46, 1.29)

*^a^Stratified analysis was performed by grouped vitamin E concentrations in the first trimester; In each group, the association of gestational change with pre-eclampsia was assessed by using robust Poisson regression models that adjusted for age, education, ethnic origin, geographical region, Hukou, ART, primigravida, pre pregnancy BMI groups, and diabetes.*

*^b^The model additionally adjusted for age, education, ethnic origin, geographical region, Hukou, ART, primigravida, pre pregnancy BMI groups, diabetes, grouped vitamin E concentrations in the first trimester, and an interaction term of grouped vitamin E concentrations in the first trimester and the gestational change.*

*Bold number refers to the value of RRs with 95%CIs significantly lower than 1.*

## Discussion

To our knowledge, this is the largest cohort study to document the association of vitamin E concentrations in the first trimester and the relative change during pregnancy with pre-eclampsia. Unified, well-established, and validated measures for gestational vitamin E determination and pre-eclampsia assessment reduced information bias, which is also indicated by the consistency of our estimates on incidence of pre-eclampsia and vitamin E deficiency with previous findings (6, 32). Our findings lend further support that prenatal vitamin E status may play an important role in pre-eclampsia.

We revealed a non-linear “L-shaped” association: the lowest incidence was observed in women with serum vitamin E concentration of 7.3–11.4 mg/L in the first trimester; serum vitamin E concentration less than 7.3 mg/L in the first trimester was associated with sharply increased risk of pre-eclampsia; above this threshold, higher vitamin E concentrations did not confer additional benefit, suggesting a “ceiling effect.” This L-shaped association has never been reported by previous studies, although several case-control studies (19, 36, 37) and cohort studies (38) found that low vitamin E levels were associated with increased lipid peroxidation product malonaldehyde levels and risk of pre-eclampsia. These existing studies failed to identify the different effects of vitamin levels in varied gestational period or relative change during pregnancy.

There has been little quantitative analysis on the association of gestational vitamin E change with the risk of pre-eclampsia. In common pregnant women, serum vitamin E concentrations progressively increase by about 40% during pregnancy (32). Our data demonstrate a L-shaped association of gestational vitamin E change with the risk of pre-eclampsia given vitamin E concentrations in the first trimester. Significantly higher risk of pre-eclampsia was found in those with decreased vitamin E concentrations during pregnancy, although gestational vitamin E increase did not confer additional benefits for those with vitamin E concentrations of ≥ 7.3 mg/L in the first trimester. Alarmingly high risk of pre-eclampsia was found in pregnant women with low vitamin E concentration in the first trimester and the decreased concentration during pregnancy.

Our findings may explain why vitamin E supplementation was ineffective in preventing pre-eclampsia in most RCTs (23, 25–31). Although vitamin levels prior to supplementation were not reported, these studies are likely to have been done in pregnant women with adequate baseline antioxidant status, considering that most of the trials were conducted in developed countries. Moreover, several RCTs found no benefit of vitamin E supplementation among women with risk factors of the disorder in reducing the occurrence of pre-eclampsia (21, 24), although few studies with the opposite conclusion exist (20). In a RCT conducted among women with type 1 diabetes, subgroup analysis found that vitamin C and E supplement reduced the risk of pre-eclampsia in women with plasma ascorbate < 10 μmol/L or serum α-tocopherol ≤ 5 μmol/mmol cholesterol (26). Similarly, our study found that only among women with the vitamin concentration of < 7.3 mg/L in the first trimester, gestational vitamin E increase was associated with the risk reduction of pre-eclampsia. These findings suggest that the beneficial effect of vitamin supplementation may be limited to women with low antioxidant status.

According to our findings, pregnant women with lower vitamin E concentration in the first trimester and those with decrease of vitamin E level during pregnancy are at high risk for pre-eclampsia. Among participants of this study, 5% had vitamin E concentration of lower than 7.3 mg/L in the first trimester and more than 10% had decreased vitamin E concentration during pregnancy from the first trimester. The estimates may represent the status of whole pregnant women in China, considering the multi-center and large-sample characteristics. In some low- and middle-income countries, the rate of vitamin E deficiency may be higher among pregnant women (39). Our study suggests that adequate vitamin E concentrations could reduce the risk of pre-eclampsia. More randomized controlled trials can be conducted in mothers with low baseline antioxidant status rather than common pregnant women to examine the effect of vitamin E supplementation in preventing pre-eclampsia.

Limitations exist in this study. Despite controlling many socio-demographic factors and pregnancy complications in multivariable adjusted analyses, we failed to measure several important factors that may confound the observed associations, such as smoking, nutrition habits, other nutrient intakes, physical activity, ingesting aspirin-like compounds, and information regarding the previous pre-eclampsia and other related complications. Additionally, due to the nature of observational research, we still do not rule out the possibility that both pre-eclampsia and vitamin E levels during pregnancy may be the result from a certain inducement, rather than a causal relationship between them. Randomized controlled trials and mechanism research are therefore warranted in the future.

## Conclusion

In conclusion, we demonstrated an L-shaped association of vitamin E status in the first trimester and the gestational change with pre-eclampsia risk, suggesting both a threshold and a ceiling effect of vitamin E. the lowest risk of pre-eclampsia was observed in women with serum vitamin E concentration of 7.3–11.4 mg/L in the first trimester. Serum vitamin E concentrations of < 7.3 mg/L in the first trimester and vitamin E decrease during pregnancy can shapely increase pre-eclampsia risk; conversely, vitamin E increase during pregnancy can mitigate the detrimental effects among only women with the vitamin concentration of < 7 mg/L in the first trimester. These findings underscore the need to supplement antioxidant vitamins among women with low baseline antioxidant status rather than common pregnant women; however, this idea needs confirmation by RCTs. More importantly, because extensive determination of vitamin E in common pregnant women is not recommended, further work is warranted to explore acceptable and cost-effective methods for identifying such high-risk women.

## Data Availability Statement

Data described in the manuscript, code book, and analytic code will be made available upon request pending application and approval by YWe, weiyuanbysy@163.com.

## Ethics Statement

The studies involving human participants were reviewed and approved by Peking University Third Hospital Medical Science Research Ethics Committee. The patients/participants provided their written informed consent to participate in this study.

## Author Contributions

YWe had full access to all of the data in this study and takes responsibility for the integrity of the data and the accuracy of the data analysis. HS and YWe: concept and design. HS, YJ, PY, LC, XG, YY, YWa, HJ, YL, MS, YZ, and YWe: acquisition, analysis, or interpretation of data and critical revision of the manuscript for important intellectual content. HS, YJ, PY, and LC: drafting of the manuscript. HS and YWa: statistical analysis. YZ and YWe: obtained funding, administrative, technical, or material support, and supervision. All authors reviewed and approved the final manuscript. The corresponding authors attests that all listed authors meet authorship criteria and that no others meeting the criteria have been omitted.

## Conflict of Interest

The authors declare that the research was conducted in the absence of any commercial or financial relationships that could be construed as a potential conflict of interest.

## Publisher’s Note

All claims expressed in this article are solely those of the authors and do not necessarily represent those of their affiliated organizations, or those of the publisher, the editors and the reviewers. Any product that may be evaluated in this article, or claim that may be made by its manufacturer, is not guaranteed or endorsed by the publisher.
