# Supplementary material for: Association of Gestational Vitamin E Status With Pre-eclampsia: A Retrospective, Multicenter Cohort Study
Source: Front Nutr. 2022 Jun 21;9:911337. doi: 10.3389/fnut.2022.911337 (PMC9253635; doi:10.3389/fnut.2022.911337)
Supplement: Supplementary file 1 [file Table_1.DOC]

**Association of Gestational Vitamin E status with Pre-eclampsia: A Retrospective, Multicenter Cohort Study**

**Supplementary Material**

**
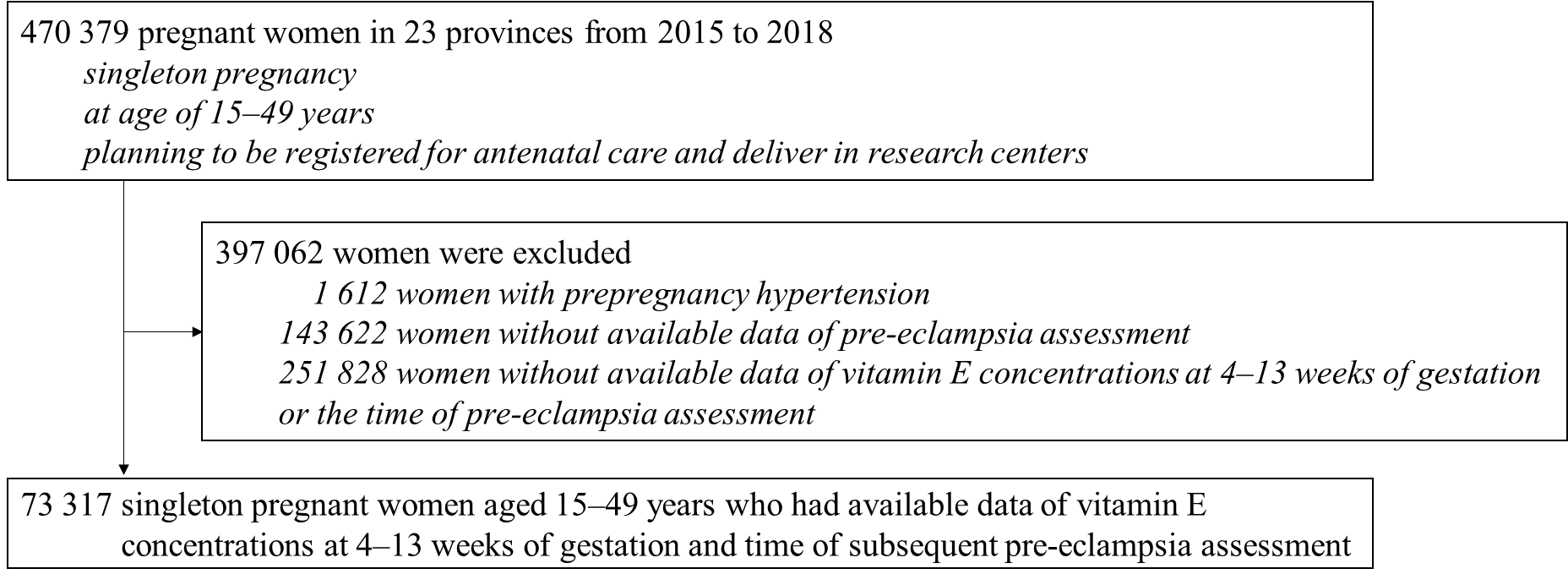
**

**Figure S1 Study profile**

**Table S1 Serum vitamin A and E concentrations (mg/L) during pregnancy in the study population**

|  |  |  | **Percentiles** | | | | | | | | | | |
| --- | --- | --- | --- | --- | --- | --- | --- | --- | --- | --- | --- | --- | --- |
|  | **N** | **Mean** | **1th** | **2.5th** | **5th** | **10th** | **25th** | **50th** | **75th** | **90th** | **95th** | **97.5th** | **99th** |
| **Vitamin E concentrations (mg/L)** |  |  |  |  |  |  |  |  |  |  |  |  |  |
| At 4–13 weeks (For all women with available data) | 219616 | 12.08 | 5.50 | 6.50 | 7.30 | 8.10 | 9.60 | 11.50 | 14.00 | 16.90 | 19.00 | 21.10 | 23.90 |
| At 4–13 weeks (For women included in this analysis) | 73317 | 11.59 | 5.37 | 6.34 | 7.20 | 8.00 | 9.30 | 11.00 | 13.30 | 16.00 | 17.90 | 19.70 | 22.10 |
| At the time of pre-eclampsia assessment | 73317 | 14.97 | 5.60 | 7.40 | 8.70 | 10.00 | 12.20 | 14.70 | 17.43 | 20.10 | 22.10 | 24.10 | 26.60 |
| Relative change during pregnancy (%) | 73317 | 36.10 | -50.61 | -37.70 | -25.97 | -12.00 | 2.86 | 31.67 | 62.29 | 92.98 | 114.84 | 134.65 | 157.74 |

**
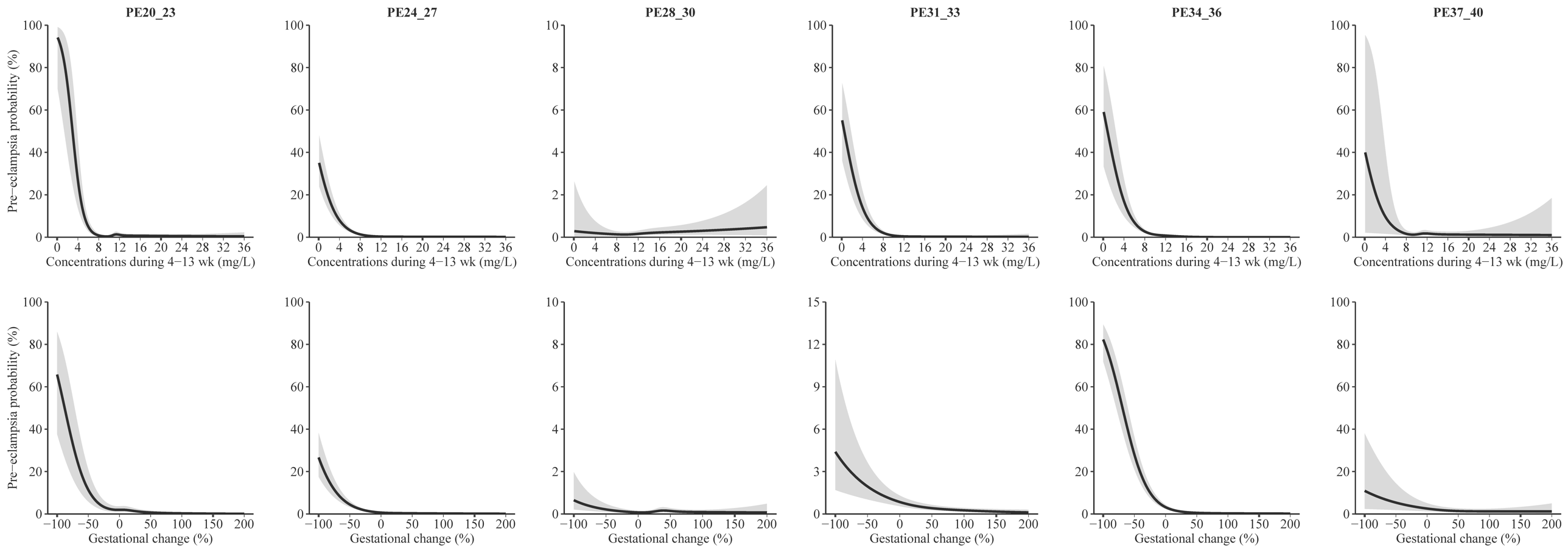
**

**Figure S2 Estimated absolute risks of pre-eclampsia with respect to vitamin E concentrations in the first trimester and gestational change during pregnancy**
